# The Role of Inflammatory Proteins in Anti-Glucocorticoid Therapy for Treatment-Resistant Depression

**DOI:** 10.3390/jcm10040784

**Published:** 2021-02-16

**Authors:** Rebecca Strawbridge, Alzbeta Jamieson, John Hodsoll, Ian Nicol Ferrier, Richard Hamish McAllister-Williams, Timothy R. Powell, Allan H. Young, Anthony J. Cleare, Stuart Watson

**Affiliations:** 1 Department of Psychological Medicine, Institute of Psychiatry, Psychology & Neuroscience, PO74, King’s College London, De Crespigny Park, London SE5 8AZ, UK; Alzbeta.Labusova@seznam.cz (A.J.); allan.young@kcl.ac.uk (A.H.Y.); anthony.cleare@kcl.ac.uk (A.J.C.); 2 National Institute for Health Research Maudsley Biomedical Research Centre, South London & Maudsley NHS Foundation Trust, London SE5 8AZ, UK; john.hodsoll@kcl.ac.uk; 3 Department of Biostatistics, Institute of Psychiatry, Psychology & Neuroscience, King’s College London, London SE5 8AF, UK; 4 Northern Centre for Mood Disorders, Translational and Clinical Research Institute, Newcastle University, Newcastle NE4 5PL, UK; nicol.ferrier@newcastle.ac.uk (I.N.F.); Hamish.mcallister-williams@newcastle.ac.uk (R.H.M.-W.); stuart.watson@newcastle.ac.uk (S.W.); 5 Cumbria, Northumberland, Tyne and Wear NHS Foundation Trust, Newcastle NE4 5PL, UK; 6 Social, Genetic & Developmental Psychiatry Centre, Institute of Psychiatry, Psychology & Neuroscience, King’s College London, London SE5 8AF, UK; timothy.1.powell@kcl.ac.uk

**Keywords:** depression, inflammation, metyrapone, glucocorticoid, augmentation, treatment-resistance

## Abstract

Background: Optimising treatments for patients with treatment-resistant depression (TRD) is key to reducing the burden of this severe illness. The anti-glucocorticoid medication metyrapone has mixed evidence supporting a role as a possible augmentation treatment in TRD. The degree of treatment resistance in depression has been associated prospectively and retrospectively with elevated inflammation, and inflammatory activity may influence responses to antidepressant treatments. Aims: To investigate whether levels of pro-inflammatory cytokines are associated with clinical outcomes to metyrapone or placebo. Methods: A double-blind RCT randomised patients with TRD to 3 weeks of placebo or metyrapone augmentation to ongoing serotonergic antidepressants. No benefit of metyrapone was reported in the primary analysis. The current study assessed levels of pro-inflammatory proteins interleukin-6 (IL-6), tumour necrosis factor (TNFα), c-reactive protein (CRP) and interleukin-10 (IL-10) before randomisation and after treatment as potential moderators and/or mediators of clinical outcomes. Results: The three pro-inflammatory proteins (but not IL-10) were elevated in this sample of patients with TRD compared to a non-affected control group. High pre-treatment IL-6 levels predicted a poorer response in the trial overall but did not moderate response to metyrapone versus placebo. Changes in IL-6 indirectly mediated depression outcome, with metyrapone increasing IL-6 levels and IL-6 increase associated with a poorer outcome on depression. Other inflammatory proteins did not mediate or moderate treatment outcomes. Interpretation: Metyrapone is hypothesised to have a therapeutic effect in depression on the basis of inhibiting the synthesis of cortisol. In this study, metyrapone did not reduce cortisol, possibly due to glucocorticoid system overcompensation). The mediation effect of IL-6 may support this and perhaps help to indicate why the treatment was not effective.

## 1. Introduction

Treatment-resistant depression (TRD) contributes substantially to the burden of depression [1]. Current drug strategies for TRD utilise serendipitously discovered treatment options and medications borrowed from other neuropsychiatric conditions with often inadequate evidence bases [2]. The hope for new drug strategies that target the identified pathophysiology, particularly dysregulation of the hypothalamic-pituitary-adrenal (HPA) axis has, as yet, failed to impact clinical practice [3]. The HPA axis mediates endocrine responses to stress by controlling the secretion of glucocorticoids such as cortisol [4]. Glucocorticoids act on multiple target tissues, affecting peripheral functions [5]. HPA axis dysregulation, notably glucocorticoid resistance (which results in chronically elevated levels and reactivity of cortisol), is commonly found in TRD and appears to play an important role in the underlying pathophysiology [6]. It is naïve, however, to consider the HPA axis in isolation. Glucocorticoid resistance and other aspects of the HPA axis function are closely related to inflammation [7]. There is now mounting evidence showing that cytokine-mediated inflammatory processes are important for the development and maintenance of depressive disorders. Many depressed patients, for instance, show the crucial features of an inflammatory response (including elevated levels of cytokine proteins) [8,9]. Inflammatory responses may be particularly heightened in those with TRD [10,11]. Elevated levels of interleukin-6 (IL-6), c-reactive protein (CRP) and tumour necrosis factor (TNFα) have been found to ‘sabotage or circumvent’ many of the mechanisms of action of conventional antidepressants [12] with high pre-treatment levels predicting non-response [13]. Whilst this phenomenon is not always consistent across studies or, for most inflammatory proteins, at a meta-analytic level [13], elevated IL-6 has preceded a poorer response in studies of patients with TRD, e.g., to N-acetylcysteine [14] or multidisciplinary inpatient treatment [10]. Thus, aberrant HPA axis and inflammatory system processes may constitute important pathophysiological processes in depression [15], with the resulting chronically elevated levels of glucocorticoids and cytokines representing biomarkers linked to treatment resistance or poor prognosis [6,9,13,16]. Influential clinical trials using HPA and immune agents include a broadly positive study of the cortisol synthesis inhibitor metyrapone in an inpatient sample with TRD [17] and a study showing a positive response using the TNFα monoclonal antibody drug, infliximab, in those TRD participants with elevated baseline immune markers [18].

In the largest anti-glucocorticoid TRD trial to date, the Anti-glucocorticoid augmentation of anti-Depressants in Depression (ADD) study, metyrapone did not reduce depressive symptoms compared with placebo, nor did it cause a sustained change in salivary cortisol levels [19]. Here we report immune data from this study, in order to examine the relationship between immune parameters, HPA axis function and clinical response to metyrapone in participants with TRD. 

The primary objective of this analysis is to evaluate associations between levels of pro-inflammatory markers; specifically IL-6, CRP, and TNFα, and the anti-inflammatory cytokine IL-10, with a therapeutic response to metyrapone or placebo augmentation of serotonergic antidepressant medication in TRD patients in order to explore whether these inflammatory proteins influenced the extent of symptom change. To do so, we examined whether:

(a) baseline inflammatory protein levels represented predictors or moderators of clinical improvement following treatment with metyrapone versus placebo;
(b) changes in inflammatory proteins represented indirect mediators of response (i.e., whether treatment allocated affected cytokine levels, and whether cytokine changes were associated with clinical outcomes).

## 2. Methods

### 2.1. Design

The ADD study was a multi-centre, two-arm parallel-group, double-blind, randomised, placebo-controlled trial. The trial design and procedures have been detailed previously [19,20]. Ethical approval was granted by the Sunderland Local Research Ethics Committee (REC reference number 10/H0904/9, 22/04/2010) and the trial was registered (EudraCT reference 2009-015165-31). All participants provided written informed consent.

### 2.2. Participants

Inclusion criteria included people aged 18 to 65 years, with a DSM-IV diagnosis of major depressive episode non-responsive to >2 antidepressants during the current episode, current moderate or severe symptoms of depression (score >18 on the 17-item Hamilton Depression Rating Scale (HAM-D [21]) and current prescription of a monotherapeutic or combination antidepressant treatment that included a serotonergic drug taken at a stable dose for >4 weeks. Patients were excluded if they had a comorbid DSM-IV axis I diagnosis (except anxiety secondary to TRD), substance use disorder, contraindication to metyrapone, were pregnant or breastfeeding or had recently participated in research that could influence results. A cohort of 31 age- and gender-matched individuals served as a control group for comparison of baseline inflammatory marker levels. These control participants had no current or lifetime axis I disorder according to DSM-IV criteria [22], no first-degree family history of mental illness, and had a HAM-D score <5. All participants were retrospectively excluded if they had a medical condition likely to influence inflammatory marker levels, had recently undergone surgery, or if they were on medication likely to influence inflammatory marker levels (e.g., oral contraception).

### 2.3. Procedures 

As detailed previously [19,20], participants continued existing antidepressant treatments and received study drug (identical capsules of metyrapone 500 mg or placebo) twice daily for 21 days. Biological and non-biological measures were administered at week 0 (prior to randomisation) and week 5 (primary outcome assessment, two weeks following discontinuation of study drug). In those randomised to metyrapone, an increase in 11-deoxycortisol was used as a measure of adherence [19]. 

### 2.4. Measures 

The severity of depressive symptoms was measured using the Montgomery–Åsberg Depression Rating Scale (MADRS [23]) administered by blinded raters at week 0 (baseline) and week 5 (primary outcome timepoint). MADRS is the primary outcome measure and we use the term ‘treatment outcome’ or ‘clinical outcome’ henceforth when denoting the week 5 MADRS score as an outcome measure while adjusting for the week 0 MADRS score as a covariate. Adverse childhood experiences were evaluated before randomisation, using the Childhood Trauma Questionnaire (CTQ [24]). 

Blood samples were taken to capture circulating levels of IL-6, TNFα, CRP and IL-10 in serum with collection, processing and quantification details as published previously [25]. Peripheral blood samples were assayed using multiplex ELISA-derived electrochemiluminescence techniques with samples randomised across multiple assay batches, with high reliability and a high agreement between the concentration and fluorescence signal (coefficient of determination r = 0.99), providing high-sensitivity measures of proteins in serum (Meso Scale Discovery V-PLEX, Meso Scale Diagnostics, USA) [25].

### 2.5. Data Analyses 

Descriptive statistics evaluated the distributions of cytokine data before and after base 10 logarithmic transformation was performed. 

#### 2.5.1. Baseline Inflammation Comparisons

Binary regressions tested baseline levels of the respective inflammatory markers, gender distribution, age, BMI and CTQ score between patients at baseline and controls. To assess the missing at random assumption, baseline predictors of missing MADRS week 5 datapoints were entered into a logistic regression and predictors of missingness at *p* < 0.01 were included in the main analysis models. All regressions included a bootstrap of 5000 resamples.

#### 2.5.2. Preliminary Outcome Prediction

Linear regression analyses were undertaken to identify whether each baseline inflammatory marker (independent variable) predicted subsequent clinical outcome (week 5 MADRS as dependent variable with week 0 MADRS as covariate) irrespective of treatments taken during the trial.

Moderation and mediation analyses were conducted in R version 3.6.1 employing a structural equation modelling (SEM) framework, using full maximum likelihood to handle missing data. 

#### 2.5.3. Moderation Analysis

To examine whether baseline protein levels served as a moderators of treatment outcome to metyrapone versus placebo, moderation analyses assessed treatment arm and protein level entered as fixed effects. Their interaction examined each variable’s relative prediction of treatment outcome (with week 5 MADRS as dependent variable and week 0 MADRS as covariate). 

#### 2.5.4. Mediation Analysis

**Figure 1:**
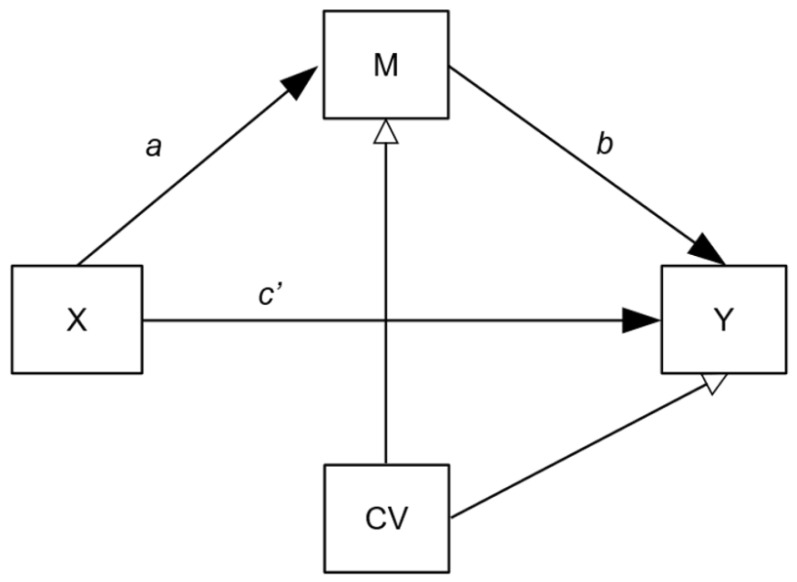
Mediation model pathway diagram. Mediation models assessing potential mediation effect of inflammatory protein changes on the relationship between randomisation group (metyrapone versus placebo) and treatment outcome: X = randomisation group (metyrapone or placebo), M = week 5 inflammatory marker (mediator), Y = week 5 MADRS (depression severity), CVs = week 0 inflammatory marker, week 0 depression severity, age, gender, BMI, CTQ. *a* path = effect of treatment on protein (week 5 adjusted for week 0), *b* path = effect of protein (week 5 adjusted for week 0) on depression severity (week 5 adjusted for week 0), *c′* path direct effect of treatment on clinical outcome.

These regression models assessed whether inflammatory protein (after treatment, adjusted for baseline levels) mediated the relationship between treatment (metyrapone versus placebo) and treatment outcome (MADRS score after treatment, adjusted for baseline levels). The mediation model is depicted in Figure 1. Due to the asymmetry of the distribution of moderator and mediation parameters, we based inference on bootstrapping 95% confidence intervals [26]. Considerations were made over the role of two potentially important factors:

(1) Confounding: Mediation models are potentially susceptible to hidden confounding on the *b* pathway and so we adjusted for age, gender, BMI and CTQ scores.
(2) Treatment adherence: Per protocol, the data of patients randomised into the metyrapone treatment arm who had not been adherent to treatment (defined as per ADD study protocols [19,20]) were excluded from the main moderation/mediation analyses, since this project’s objective was to consider the confluences of inflammation and metyrapone treatment. However, this exclusion could introduce selection bias as non-adherers are removed from the intervention group only. With this in mind, we also present a modified intention-to-treat (mITT) analysis as a secondary outcome, in which all randomised participants (except where acute inflammation was indicated; see below) were included.

## 3. Results

*n* = 174 individuals were identified from the ADD trial (*n* = 143 patients; *n* = 31 controls) whose inflammatory and clinical data were available. Of these, 17 were retrospectively excluded from analysis (seven patients due to inflammatory condition or medication; ten due to substantially elevated inflammatory marker levels indicating possible infection). Statistical analysis was therefore carried out using the data of 129 patients (placebo *n* = 66; metyrapone *n* = 63) and 28 control participants (see Table S1 for further detail including adherence and follow-up rates). 

Of the 129 participants, 23/63 randomised to metyrapone—over one third—did not adhere to treatment. The 40 adherent participants are included in primary analyses, alongside all 66 randomised to placebo (as their adherence could not be determined). Inflammatory protein data was complete (no protein levels undetected). 

### 3.1. Participant Characteristics

Baseline characteristics of patients and controls are shown in Table 1. Gender distribution and age were comparable between groups, while control participants had a lower adverse childhood experience severity (*t*(82) = 6.28 [95% CI 12.17, 23.47], *p* < 0.001) and BMI (*t*(153) = 4.28 [95% CI 2.95, 8.03], *p* < 0.001) than patients. Comparable gender, age, BMI and childhood adversity scores were present between participants randomised to metyrapone versus placebo. Baseline differences in inflammatory markers between metyrapone and placebo groups were also negligible. In the full sample, age was positively associated with IL-6 (*r* = 0.22, *p* = 0.005) and BMI was positively associated with all three pro-inflammatory markers (IL-6 *r* = 0.56, *p* < 0.001; TNFα *r* = 0.25, *p* = 0.002; CRP *r* = 0.57, *p* < 0.001), while childhood adversity was not associated with any of the studied inflammatory markers.

### 3.2. Baseline Inflammation Comparisons 

Pro-inflammatory proteins at baseline were significantly higher in patients than controls after adjustment for gender, age, childhood adversity and BMI: independent effects of markers elevated in TRD patients: IL-6 X^2^ = 0.061 [95% CI 0.005, 0.719], *p* = 0.026; TNFα X^2^ = 0.005 [95% CI 0.0009, 0.554], *p* = 0.028; CRP X^2^ = 0.119 [95% CI 0.027, 0.526], *p* = 0.005. The anti-inflammatory cytokine IL-10 was not significantly different between groups. Multicollinearity within regression models was not significant. Descriptive statistics can be found in Table 2 and a depiction of patient/control differences in Figure S1. 

A total of 27% of the variance in post-treatment depression severity (MADRS score) was explained by a model comprising randomisation group (non-significant) and baseline depression severity (β coefficient = 0.93 [95% CI 0.67, 1.20], *p* < 0.001). The model R^2^ value increased from 0.27 to 0.31 when including IL-6, with higher baseline IL-6 associated with a poorer treatment outcome (week 5 MADRS adjusted for week 0 MADRS); β coefficient = 3.31 [95% CI 0.24, 6.46], *p* = 0.034. Other cytokines were not associated with subsequent outcome across the sample (*p* > 0.1). These results are presented in Table 3; the association between pre-treatment IL-6 and subsequent outcome is also presented in Figure S2A. The model R^2^ increased to 0.33 when including all factors in a single model, with only baseline depression severity contributing significantly (*p* < 0.001).

### 3.3. Inflammatory Markers as Potential Moderators of Clinical Outcomes 

None of the inflammatory proteins at baseline significantly moderated treatment outcome to metyrapone compared with placebo (see Table 3). In the mITT analysis, TNFα showed a non-significant interaction with group, with higher levels associated with more severe depression at post-treatment in metyrapone than placebo groups (β = 8.24 [95% CI −1.59, 18.01], standardised effect size (SES) = 0.36, *p* = 0.097). 

### 3.4. Inflammatory Markers as Potential Mediators of Treatment Effects 

**Figure 2:**
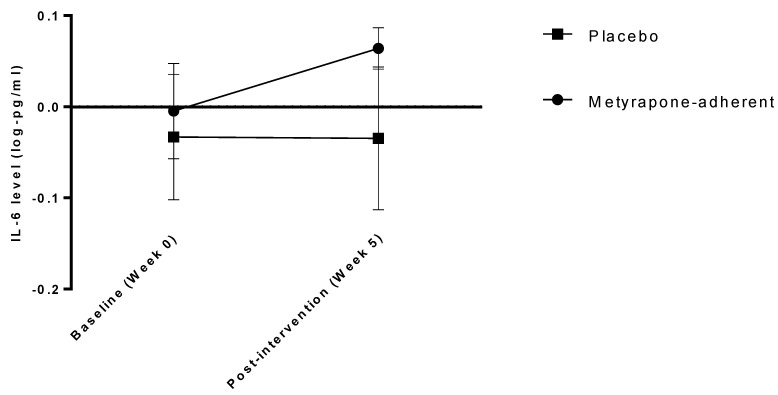
IL-6 levels at baseline and after treatment (five weeks after randomisation) in participants taking placebo and metyrapone. Log-transformed mean values of interleukin 6 (IL-6) before and after treatment in the metyrapone group (significant increase over time) and placebo group (no significant change over time). Error bars represent standard deviation (SD).

When testing the *a* path with the treatment group (independent variable), baseline protein (covariate) and post-treatment protein (dependent variable) included in a regression model, no significant effects were indicated for IL-10 (β = 0.22 [95% CI −0.05, 0.50], *p* = 0.114), CRP (β = 0.08 [95% CI −0.23, 0.44], *p* = 0.631) or TNFα (β = 0.04 [95% CI −0.12, 0.24], *p* = 0.654) but indicated a significant effect of treatment on IL-6 (β = 0.37 [95% CI 0.14, 0.61], *p* = 0.002; see Figure 2). A full mediation model was therefore explored for this protein, as presented in Table 4. IL-6 appears to partially mediate an association between treatment and outcome: participants taking metyrapone—but not placebo—had increased IL-6 after treatment (coefficient *a path* = 0.38 [95% CI 0.14, 0.66], SES = 0.24) and a greater increase in IL-6 was associated with a poorer clinical outcome after treatment (coefficient *b path* = 3.12 [95% CI 0.43, 6.29], SES = 0.23); the association between IL-6 change and clinical outcome is also presented in Figure S2B. The indirect effect of treatment group on severity after treatment indicated a significant effect of IL-6 (*ab* coefficient = 1.20 [95% CI 0.18, 3.41], SES = 0.06). There was no direct effect of treatment group on clinical outcome (regression coefficient *c’ path* = −1.92 [95% CI −6.03, 2.19], SES = −0.09). When including all randomised participants in this mediation model (mITT), the overall indirect effect was weaker, just missing significance at *p* ≤ 0.05; *ab* coefficient = 0.69 [95% CI −0.01 to 2.36], SES = 0.03.

## 4. Discussion

Patients with TRD in this study demonstrated signs of elevated inflammation. Concentrations of the cytokines TNFα, CRP and IL-10 were not influenced by metyrapone treatment and were not predictive of clinical response (either baseline levels or changes during treatment) in the ADD trial. Conversely, IL-6 appears to be implicated, potentially in multiple ways. While not differing between metyrapone and placebo groups, participants with higher pre-treatment IL-6 tended to have more severe depression at the end of the trial. Furthermore, metyrapone treatment appeared to actively increase levels of IL-6, with this increase associated with more severe depression after the intervention period. 

The ADD study was conducted in response to evidence showing potential for anti-glucocorticoid antidepressant benefits, particularly for the cortisol inhibitor metyrapone [17,28,29]. The ADD study was the only adequately powered, randomised study of metyrapone augmentation for patients with established TRD. The negative findings in this study which contrasted with previous studies may be attributed to various factors such as the tendency of unblinded or small studies to yield-inflated effect sizes [30], a potentially insufficient treatment duration [2], the population (established TRD is by definition a challenge to treat and participants in the ADD study demonstrated a high TRD severity overall in contrast to many RCTs recruiting patients ostensibly with TRD but not severely enough to meet common clinical definitions [2]) or the absence of baseline hypercortisolemia. 

It is noteworthy that salivary cortisol was not affected by metyrapone at the primary endpoint. Rather than successfully lowering cortisol through inhibiting its synthesis, metyrapone may have caused a compensatory increase in HPA axis activity with an homeostatically driven increase in ACTH which served to maintain cortisol concentrations [19]. Peripheral IL-6 levels were elevated at week 5 compared with week 0. Glucocorticoid elevations typically suppress pro-inflammatory cytokines (prominently IL-6); the increase in IL-6 after metyrapone treatment might suggest a freeing from glucocorticoid control of pre-existing inflammation. IL-6 has been reliably demonstrated to exert acute stimulatory effects on adrenal functions and, when organisms are immunologically challenged, to activate the HPA axis independently of corticotropin-releasing hormone increases [31,32]. Therefore, the lack of decrease in salivary cortisol concentrations at the primary outcome timepoint (two weeks after treatment cessation) [19] could have been partly driven by the increased IL-6, and this lack of a corticosteroid-lowering effect combined with a direct effect of inflammatory processes on mood, [e.g., 8,11] may have contributed to the lack of a clinical effect on symptoms.

The duration of treatment was selected to be 3 weeks following concerns over longer-term treatment with anti-glucocorticoids and the precedent of previous evidence indicating this as an apposite duration [17]. The primary outcome was two weeks afterwards because evidence suggests that clinical and biological (HPA axis) effects persist for at least two weeks after treatment discontinuation, as supported by Jahn et al. [17] who employed the same outcome timepoints. In spite of these previous indications, it may be that our inflammatory findings would nevertheless have differed if measured at week 3. For example, TNFα, CRP and IL-10 may have been altered by metyrapone at week 3 and/or demonstrated mediation effects on subsequent outcomes, having been more directly influenced by trial interventions. Likewise, IL-6 may not have increased until after metyrapone cessation (e.g., rebound effect). However, ACTH was raised markedly by metyrapone at week 3 [19] and this might be expected to reflect increased pro-inflammatory immune markers, particularly IL-6 [5]. Additional measurement of inflammatory outcomes at week 3 would clarify the temporal influences of metyrapone on inflammatory proteins, although arguably in this case the clinical significance of findings may be of more interest in the weeks following treatment cessation than at discontinuation.

It is worth noting that the melancholic subtype of depression has been purported to present with hypercortisolemia but with lower inflammation than the atypical subtype, which has been associated with inflammatory elevations but normal (or in some cases even attenuated) adrenal activity [33]. The ‘atypical’ profile of ADD participants was not recorded. Anti-glucocorticoid medications may therefore be more suited to those with melancholic depression.

HPA dysregulation predicts better clinical response to anti-glucocorticoids in bipolar [34] and unipolar [35] depression, and elevated inflammatory markers predict response to anti-inflammatory drugs [18]. The interconnectedness of HPA and inflammatory processes may suggest that examination of both baseline HPA and inflammatory markers together may improve the ability to predict a positive response to anti-glucocorticoid and/or anti-inflammatory agents and a lack of response to standard pharmacological treatments [6,16,36].

The findings reported in this study should be interpreted cautiously for a number of reasons. Firstly, participants who did not adhere to metyrapone were excluded from the main mediation and moderation analyses, leaving a smaller sample size. This exclusion was due to our intention to examine the effects of undertaking treatment on the protein markers; the IL-6 mediation finding cannot be clearly generalised to non-adherent individuals as shown in Table 4. Reasons for non-adherence could have been associated with inflammatory effects (e.g., via tolerability difficulties) that could not be examined, and adherence to placebo was also not ascertainable. The other participants who were excluded from the current study were those with outlier levels of inflammation that may indicate acute infection or inflammatory state. However, excluding these also may have affected findings if their aberrant inflammatory state was causally associated with subsequent clinical outcome. As we have documented previously, the proteins measured fluctuate according to a multitude of factors related to lifestyle, health and aging as well as those considered in this work [11]. Accounting for those factors in analyses is a challenge, particularly with relatively small sample sizes, and if we had attempted to do so our models would have been under-powered and more susceptible to overfitting. 

We assessed the treatment outcome at week 5 (same measurement timepoint as protein markers) but treatment effects could alternatively have been examined at week 3 (treatment endpoint) or at a longer-term follow-up timepoint. We chose to use the depression severity outcome as a continuous marker because of the low overall response/remission rate, and because using a continuous measure improves variability in models, but it is arguable that a binary variable representing ‘response’ or ‘mild/remitted severity cut-off’ could be a clinically useful binary grouping that shows clearer effects in prediction, moderation or mediation models. It is notable that the potential attributions of findings to cortisol activity are speculative and we did not assess cortisol levels in the current analyses (these are reported in the primary analysis paper [19]).

Unlike many other studies predicting outcome to augmentation trials for TRD [37] the participants in this study had established TRD, the study was robustly conducted including double blinding of investigators and patients and the present analyses provides further insights into the putative mechanisms of metyrapone treatment and the associations between inflammation and affective state in TRD. 

It would be useful to replicate this study in samples of individuals with elevated cortisol and low IL-6 prior to treatment with metyrapone and to use early change in inflammatory and HPA measures as predictors of longer-term response. This would provide information regarding precision medicine, efficacy and mechanisms related to treatment influences on inflammatory proteins (particularly IL-6) and their association with treatment outcomes. 

## 5. Conclusions

We have previously demonstrated the absence of an effect of metyrapone on cortisol levels or depression symptoms in people with treatment-resistant depression. Here, we demonstrate that baseline elevated IL-6 is a factor linked to poor prognosis and that IL-6 is increased by metyrapone. Our findings putatively suggest an explanation for the unexpected absence of influence of metyrapone on cortisol and/or the expected therapeutic response to metyrapone. Furthering our understanding of the interplay between HPA and inflammatory systems, in association with depressive symptoms and treatment effects, may be crucial in developing novel therapeutic strategies for this debilitating illness.

## Figures and Tables

**Table 1:** Baseline characteristics.

Characteristic		TRD Patients (*n* = 129)	Control Participants (*n* = 28)
Gender	Female: *n* (%)	77 (60%)	16 (57%)
	Male: *n* (%)	52 (40%)	12 (43%)
Age	Mean (SD)	47.20	43.8 (9.1)
BMI	Mean (SD)	30.88 (6.34)	25.4 (5.2)
CTQ score ^a^	Mean (SD)	52.05 (21.21)	34.2 (10.2)
MADRS score	Mean (SD)	28.09 (6.12)	n/a
IL-6 (log-pg/mL) ^a^	Mean (SD)	−0.077 (0.282)	−0.350 (0.298)
TNFα (log-pg/mL) ^a^	Mean (SD)	0.347 (0.142)	0.233 (0.120)
CRP (log-ug/mL) ^a^	Mean (SD)	6.491 (0.512)	5.908 (0.537)
IL-10 (log-pg/mL) ^a^	Mean (SD)	−0.437 (0.285)	−0.545 (0.202)

Characteristics of the sample at baseline: gender, age, BMI and IL-10 level did not differ between TRD and control participants. TRD participants randomised to metyrapone did not differ from those randomised to placebo on any baseline characteristics. ^a^ Childhood trauma severity, IL-6, TNF and CRP were higher in TRD than control participants. Log transformed values of inflammatory proteins presented. Abbreviations: TRD = treatment-resistant depression, BMI = body mass index, CTQ = childhood trauma questionnaire, MADRS = Montgomery–Åsberg Depression Rating Scale, IL-6 = interleukin 6, TNFα = tumour necrosis factor, CRP = c-reactive protein, IL-10 = interleukin 10.

**Table 2:** Symptoms and inflammation throughout the trial in metyrapone and placebo groups.

Characteristic		TRD Patients	*Metyrapone*	*Placebo*
MADRS	Week 0	28.09 (6.12)	*27.76 (6.98)* *Adherent: 27.30 (6.72)* *Not adherent: 28.57 (7.48)*	*28.39 (5.20)*
	Week 5	22.24 (11.00)	*27.72 (11.68)* *Adherent: 20.55 (10.58)* *Not adherent: 28.93 (12.81)*	*21.80 (10.43)*
IL-6 (log-pg/mL)	Week 0	−0.077 (0.282)	*−0.0511 (0.273)* *Adherent: −0.057 (0.301)* *Not adherent: −0.04 (0.222)*	*−0.102 (0.290)*
	Week 5	−0.049 (0.331)	*0.017 (0.288) * *Adherent: 0.087 (0.261)* *Not adherent: −0.148 (0.287)*	*−0.113 (0.358)*
TNFα (log-pg/mL)	Week 0	0.347 (0.142)	*0.368 (0.143)* *Adherent: 0.365 (0.142)* *Not adherent: 0.374 (0.149)*	*0.328 (0.139)*
	Week 5	0.344 (0.228)	*0.352 (0.223)* *Adherent: 0.375 (0.183)* *Not adherent: 0.298 (0.298)*	*0.337 (0.233)*
CRP (log-ug/mL)	Week 0	6.491 (0.512)	*6.485 (0.551)* *Adherent: 6.427 (0.562)* *Not adherent: 6.585 (0.528)*	*6.496 (0.475)*
	Week 5	6.465 (0.567)	*6.501 (0.549)* *Adherent: 6.428 (0.484)* *Not adherent: 6.672 (0.661)*	*6.431 (0.586)*
IL-10 (log-pg/mL)	Week 0	−0.437 (0.285)	*−0.452 (0.298)* *Adherent: −0.417 (0.284)* *Not adherent: −0.514 (0.318)*	*−0.422 (0.273)*
	Week 5	−0.393 (0.345)	*−0.379 (0.351)* *Adherent: −0.322 (0.357)* *Not adherent: −0.511 (0.308)*	*−0.407 (0.341)*

The italics indicate that the metyrapone and placebo columns are subsets of the full ‘trd patients’ sample. Characteristics of patients randomised to metyrapone versus placebo before and after treatment. Placebo *n* = 66, metyrapone *n* = 63 (adherent *n* = 40, non-adherent *n* = 23). For more details regarding missing data, see Table S1. IL-6 was higher in metyrapone participants (as a whole, randomised group and in the adherent group) at week 5 compared to week 0, and at week 5 compared to placebo-randomised participants. Log transformed values of inflammatory proteins presented. Abbreviations: TRD = treatment-resistant depression, MADRS = Montgomery–Åsberg Depression Rating Scale, IL-6 = interleukin 6, TNFα = tumour necrosis factor, CRP = c-reactive protein, IL-10 = interleukin 10.

**Table 3:** Predictive effects of inflammatory proteins on subsequent outcome to metyrapone versus placebo.

Protein	Analysis	Inflammatory Protein Predictor Effect ^a^			Interaction Effect (Protein x Group) ^b^		
		Coefficient (95% CI)	SES	*p* Value	Coefficient (95% CI)	SES	*p* Value
IL-6	Per-protocol (*n* = 106)	2.57 (−0.67, 5.96)	0.17	0.125	−3.58 (−9.61, 3.50)	−0.15	0.280
	mITT (*n* = 129)	3.31 (0.24, 6.46)	0.20	0.034 *	−2.00 (−7.74, 5.00)	−0.08	0.532
TNFα	Per-protocol (*n* = 106)	2.56 (−2.74, 7.87)	0.08	0.347	8.17 (−1.48, 19.70)	0.36	0.127
	mITT (*n* = 129)	2.56 (−2.74, 7.87)	0.08	0.347	8.24 (−1.59, 18.01)	0.36	0.097
CRP	Per-protocol (*n* = 106)	0.67 (−0.84, 2.61)	0.08	0.437	0.50 (−3.10, 4.04)	0.35	0.782
	mITT (*n* = 129)	1.14 (−0.32, 2.94)	0.12	0.176	1.16 (−2.09, 4.78)	0.80	0.508
IL-10	Per-protocol (*n* = 106)	−0.19 (−3.65, 3.26)	−0.01	0.914	−0.56 (−7.69, 6.73)	−0.10	0.880
	mITT (*n* = 129)	−0.19 (−3.65, 3.26)	−0.01	0.914	1.08 (−6.05, 8.45)	0.18	0.767

^a^ linear regression including baseline protein (independent variable), treatment group and baseline depression severity (MADRS score) (covariate) on depression outcome at week 5 (dependent variable). ^b^ regression including baseline protein, baseline depression severity, treatment group and protein x group interaction effect as potential moderator of week 5 depression outcome (dependent variable). * statistically significant at *p* 0.05. Abbreviations: SES = standardised effect size, mITT = modified intention to treat analysis, IL-6 = interleukin 6, TNFα = tumour necrosis factor, CRP = c-reactive protein, IL-10 = interleukin 10.

**Table 4:** Mediation effects of IL-6 changes on response to metyrapone versus placebo.

Comparison	Analysis	Coefficient	95% CI	SES
Effect of treatment (X) on IL-6 (mediator; M) *a path* *	Per-protocol (*n* = 106)	0.38	0.14, 0.66	0.24
	mITT (*n* = 129)	0.22	−0.03, 0.49	0.15
Effect of IL-6 (mediator; M) on outcome (Y) b path *	Per-protocol (*n* = 106)	3.12	0.43, 6.29	0.23
	mITT (*n* = 129)	3.16	0.76, 6.31	0.22
Indirect effect of treatment (X) on outcome (Y) ab *	Per-protocol (*n* = 106)	1.20	0.18, 3.41	0.06
	mITT (*n* = 129)	0.69	−0.01, 2.36	0.03
Direct effect of treatment (X) on outcome (Y) c′ path *	Per-protocol (*n* = 106)	−1.92	−6.03, 2.19	−0.09
	mITT (*n* = 129)	−0.21	−4.02, 3.50	−0.01

* Regression paths as previously named [27]. Models assessing potential mediation effect of inflammatory protein changes on the relationship between randomisation group (metyrapone versus placebo) and treatment outcome. See also Figure 1. X = randomisation group (metyrapone or placebo), M = week 5 inflammatory marker (mediator), Y = week 5 MADRS (depression severity), covariates = week 0 inflammatory marker, week 0 depression severity, age, gender, BMI, CTQ. *a* path = effect of treatment on protein (week 5 adjusted for week 0), *b* path = effect of protein (week 5 adjusted for week 0) on depression severity (week 5 adjusted for week 0), *c′* path direct effect of treatment on clinical outcome. Abbreviations: SES = standardised effect size, mITT = modified intention to treat analysis, IL-6 = interleukin 6, TNFα = tumour necrosis factor, CRP = c-reactive protein, IL-10 = interleukin 10.

## Data Availability

Please contact the corresponding author with any data availability requests.

## Supplementary Materials

The following are available online at https://www.mdpi.com/2077-0383/10/4/784/s1, Table S1: description of missing data and number of participants in all aspects of the study; Figure S1A–D showing inflammatory protein levels in TRD patients compared with unaffected controls. Figure S2A,B presenting IL-6 associations with treatment group and outcome as examined in linear regression and mediation analysis.
