# Supplementary material for: The Role of Inflammatory Proteins in Anti-Glucocorticoid Therapy for Treatment-Resistant Depression
Source: J Clin Med. 2021 Feb 16;10(4):784. doi: 10.3390/jcm10040784 (PMC7920038; doi:10.3390/jcm10040784)
Supplement: Supplementary file 1 [file jcm-10-00784-s001.pdf]

Supplementary material

Table S1: **Number of participants/missing data**

			Total TRD	Controls
Total number of ADD participants with baseline inflammatory and clinical data			**143**	31
	**Metyrapone**	**Placebo**	**Total TRD**	**Controls**
Total numbers after exclusions for inflammatory conditions [a]	63	66	**129**	28
Number after exclusion of non-adherent metyrapone patients [b]	40	66	**106**	-
Number of participants (excluding metyrapone non-adherent) with clinical and inflammatory outcome data [c]	40	57	**97**	-

[a] included in baseline patient/control comparisons, and the mITT moderation and mediation analyses
[b] included in per-protocol moderation and mediation analyses
[c] included in analyses with missing data addressed using full information maximum likelihood

Figure S1

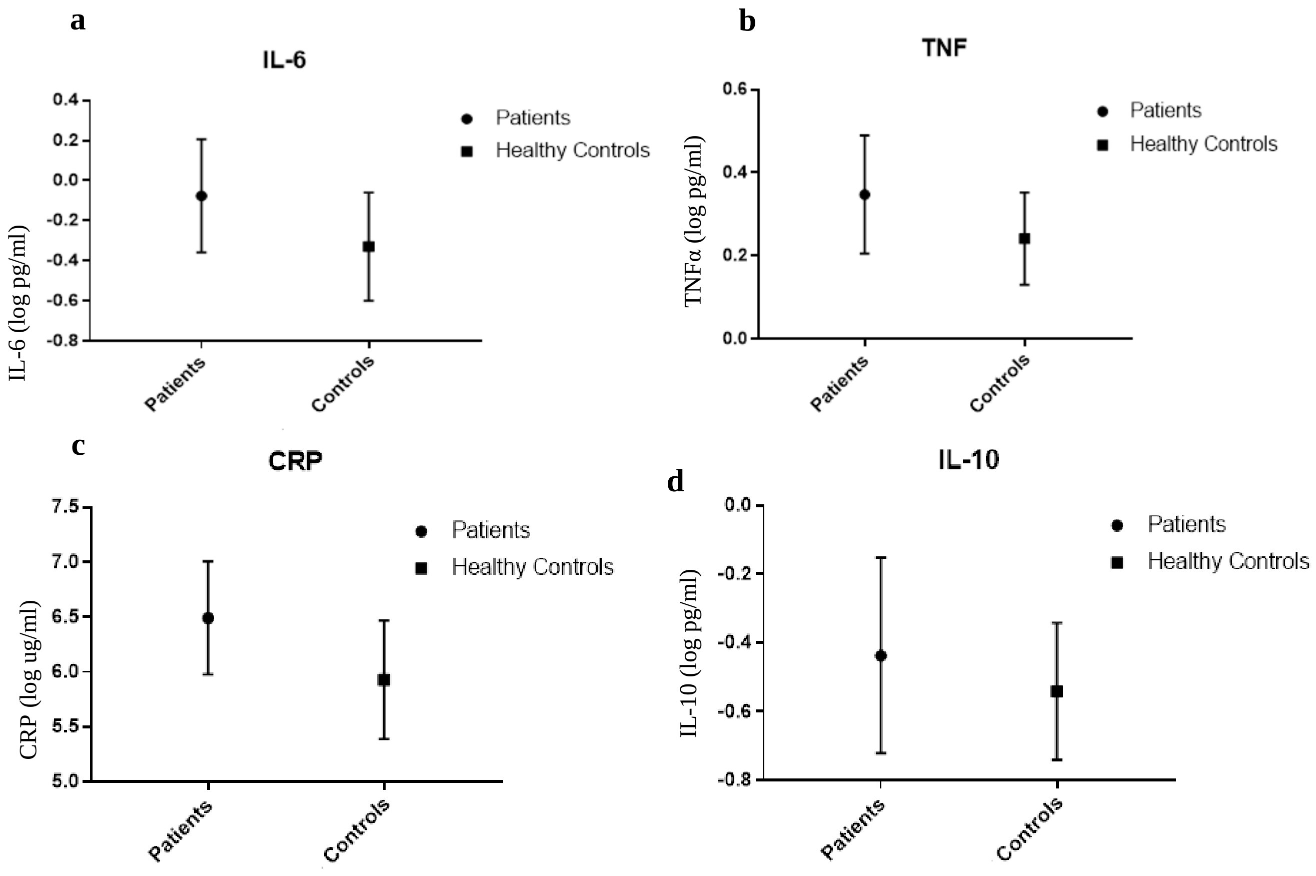

Figure a-d: Patients' and controls' baseline levels of

- **a)** IL-6
- **b)** TNFα
- **c)** CRP
- **d)** IL-10

Log transformed mean values of the cytokines assessed are presented. Error bars represent standard deviation (SD). CRP = C-reactive Protein; IL-6 = Interleukin 6; IL-10 = Interleukin 10; TNF-α = Tumour Necrosis Factor α.

Figure S2

A.

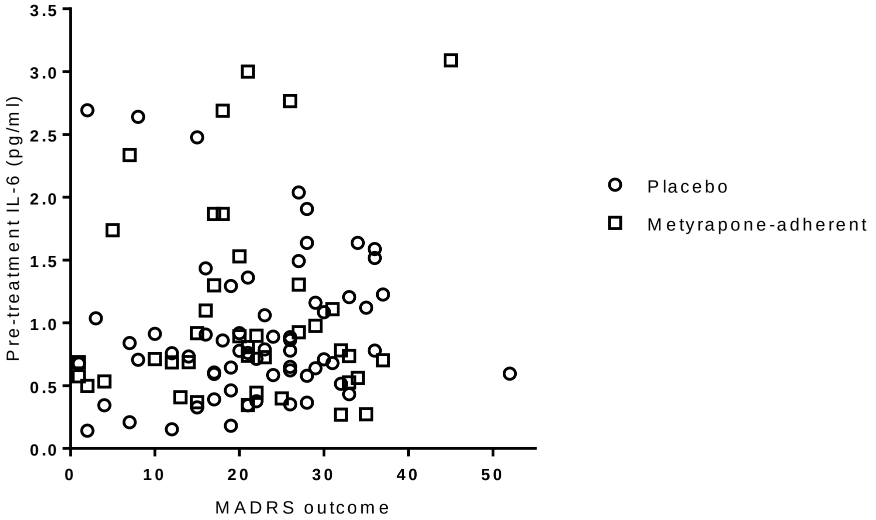

B.

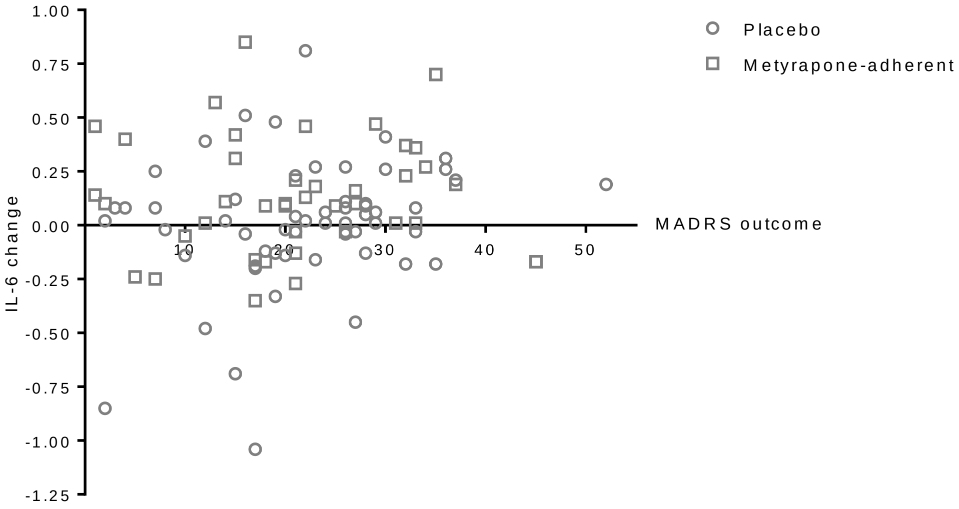

Figure A / B: IL-6 associations with treatment group and clinical outcome, as examined in linear regression and mediation analysis

a) Pre-treatment IL-6 as a predictor of subsequent clinical outcome

b) Change in IL-6 during treatment and association with clinical outcome
